# Analysis of Drug and Health Resource Use Before and After COVID-19 Lockdown in a Population Undergoing Treatment for Depression or Anxiety

**DOI:** 10.3389/fpsyg.2022.861643

**Published:** 2022-04-05

**Authors:** Ana Lear-Claveras, Ana Clavería, Sabela Couso-Viana, Patrice Nabbe, Bárbara Oliván-Blázquez

**Affiliations:** ^1^Institute for Health Research Aragón (IIS Aragón), Zaragoza, Spain; ^2^I-Saúde Group, South Galicia Health Research Institute, Vigo, Spain; ^3^Vigo Health Area, SERGAS, Vigo, Spain; ^4^Network for Research on Chronicity, Primary Care, and Health Promotion (RICAPPS), Barcelona, Spain; ^5^Département Universitaire de Médecine Générale, ER 7479 SPURBO (Soins Primaires, Santé Publique, Registre des Cancers de Bretagne Occidentale), Université de Bretagne Occidentale, Brest, France; ^6^Department of Psychology and Sociology, University of Zaragoza, Zaragoza, Spain

**Keywords:** COVID-19, depression, anxiety, quarantine, lifestyle, primary health care

## Abstract

**Introduction:**

The arrival of the COVID-19 pandemic modified the functions of Primary Care (PC) teams, which were forced to focus their resources on the diagnosis and treatment of SARS-CoV-2 infected patients. The disrupted healthcare of individuals with pre-existing mental disorders (depression or anxiety), as well as the psychological decompensation resulting from the lockdown caused by the COVID-19 pandemic, may have modified the use of drugs and health resources by these patients. The aim of this study is to determine the changes in these parameters, between the 6 months prior to the lockdown (09/14/2019 to 03/15/2020) and the 6 months following its end (05/03/2020 to 11/04/2020), in a population undergoing active treatment for depression or anxiety, according to the electronic clinical record.

**Materials and Methods:**

Real world data observational study of 110,694 individuals aged >16 years suffering from active or undergoing treatment for depression or anxiety according to the electronic medical records of the Aragon Regional Health Service (Spain). Pharmacological variables [daily dose per inhabitant (DHD) dispensed by pharmacies of: anxiolytics, hypnotics/sedatives, and antidepressants] and variables related to the use of healthcare resources (number of primary and specialized healthcare visits) were considered. Student’s *T*-tests for paired samples were performed to analyze differences between periods (pre–post). The level of significance was established at 5% (*p* < 0.05).

**Results:**

The use of anxiolytic drugs increased as compared to its use over the 6 months prior to the lockdown. In contrast, the consumption of antidepressants was found to decrease. The use of health resources continued to be below pre-pandemic levels, 6 months post-lockdown end.

**Conclusion:**

Changes in the use of health resources could have a negative impact on the parameters of these diseases. The increase in drug use, especially benzodiazepines, may suggest a worsening of the symptoms during the lockdown and in the subsequent months. It is a worrying sign, which points to the growth of this public health problem and the need for its prevention.

## Introduction

In March of 2020, the World Health Organization (WHO) declared the COVID-19 pandemic (World Health Organization (WHO), 2020a). Since then, the global population has been exposed to an endless number of stressful events. The huge threat presented by this new virus has forced numerous governments to implement restrictive measures that included limited mobility and at-home confinement. With the continued rise in infections and deaths, Spain’s government declared a national state of alarm on 15 March 2020, placing the population in an unprecedented lockdown (Gobierno de España, 2020). These restrictive measures were useful in decreasing the incidence of cases, but they had a major impact on the physical and psychological wellbeing of the population (Ramírez-Ortiz et al., 2020; Valdés-Florido et al., 2020); especially for those who suffer from pre-existing mental disorders (Sheridan Rains et al., 2021).

Despite the high global morbidity caused by these disorders, mental health continues to be one of the most neglected areas of healthcare. Many of these disorders are treated in Primary Healthcare (World Health Organization (WHO), 2018), where the prevalence of psychiatric morbidity ranges between 25 and 30% (Garcia-Toro et al., 2012) and where one out of every four patient appointments is due to these disorders (Gobierno de Canarias, 2008).

The collapse of Primary Care (PC) services at the onset of the COVID-19 pandemic may have interrupted the continuous healthcare services of these patients (World Health Organization (WHO), 2012; Kozloff et al., 2020; United Nations, 2020); and similarly, the fear of infection may have lowered the demand at healthcare centers (World Health Organization (WHO), 2020b), causing the anticipated psychopathological imbalances and an increased a posteriori demand (World Health Organization (WHO), 2012; Kozloff et al., 2020).

Social and physical distancing measures may have also had an especially negative impact on individuals suffering from mental disorders (Kozloff et al., 2020). Maintaining daily routines and social relationships is essential for the proper handling of these disorders (Druss, 2020; Kozloff et al., 2020; Sheridan Rains et al., 2021). Therefore, the interruption of these routines during the lockdown months may have exacerbated their symptoms (Fernández-Aranda et al., 2020; Zhou et al., 2020; Sheridan Rains et al., 2021). and might have favor the acquisition of unhealthy habits, which may, in turn, might have influence the physical health of these patients. In them are frequent comorbidities with other physical and also psychiatric pathologies (Firth et al., 2019).

Despite the considerable changes in daily routines and the potential decline in healthcare services provided to these patients during this crucial lockdown period, few studies have considered the impact of the COVID-19 pandemic and lockdown on the population suffering from pre-existing mental disorders (Vindegaard and Benros, 2020; Sheridan Rains et al., 2021). The few studies that have been carried out on this topic have tended to assess the short-term impact of this situation (during the peak of the pandemic) (Fernández-Aranda et al., 2020; Hao et al., 2020; Liu et al., 2020) using self-completion questionnaires (Vindegaard and Benros, 2020). The use of drugs as anxiolytics and anti-depressants as well as the consumption of health care resources are quantifiable data that can reveal behavioral change (feelings of sadness or anxiety, anhedonia, sleep disturbances, irritability, frustration, etc.) in his population. These indicators could be an indirect means of revealing the variation in psychological suffering of the population. The objective of this study is to determine and to analyze these changes, between the 6 months prior to the lockdown and the 6 months following its end, in a population undergoing active treatment for depression or anxiety, according to the electronic clinical record (ECR).

## Materials and Methods

### Design and Study Population

A real world data observational study of a population of northern Spain (Aragon) over the age of 16, undergoing active treatment for depression or anxiety, according to the ECR.

Patients having registered episodes of depression or anxiety have been included [codes F30–F39 and F41 of the 10th revision of the International Statistical Classification of Diseases and Related Health Problems (ICD-10)], as well as those who, during the study period, had been dispensed some of the active ingredients used to treat these two disorders, according to the Spanish Society of Family and Community Medicine (semFYC) (Sociedad Española de Medicina de Familia y Comunitaria [semFYC], 2020a,b). According to the anatomical, therapeutic, and chemical classification (ATC), the following analyzed codes were considered: N05B (anxiolytic drugs), N05C (hypnotics and sedatives), and N06A (antidepressants).

For each individual, information was obtained from their ECR during the two distinct time periods. The first measurement was taken from the records from the 6 months prior to the onset of the lockdown (09/14/2019 to 03/15/2020) and the second was taken from the records from the 6 months following the end of the lockdown (05/03/2020 to 11/04/2020).

The ECR is extensively consolidated in Spain and Aragon, where it was fully implemented in 2011. The data contained in it are generated throughout the healthcare process (hospital and PC) for patients seen by the National Healthcare System (90% coverage). This information is unified and available for all healthcare professionals in the system.

### Variables

Sex, age, pharmaceutical provision, and basic rural or urban health area (with more than 10,000 inhabitants), were the sociodemographic variables included in this study. For each of the measurement periods, the number of deaths in the study population was also considered; also chronic comorbidities with prevalences of over 5% were considered (Calderón-Larrañaga et al., 2017) [arrhythmias, heart failure, ischemic cardiopathy, dyslipidemia, obesity, excess weight, vein and artery disease, cerebrovascular disease, diabetes, chronic bronchitis, chronic obstructive pulmonary disease (COPD), asthma, chronic kidney disease, hypo and hyperthyroidism, tobacco use, alcoholism, insomnia, attempted suicide, anemia, neoplasia, dementia, deafness, cataracts, glaucoma, arthrosis, osteoporosis, and back pain].

Changes in drug consumption patterns have been assessed via the variation in daily dose per inhabitant (DHD) dispensed by pharmacies. Based on the defined daily dose (DDD) stipulated by the WHO, DHDs were calculated according to the formula shown below:


(1) $$\mathrm{DHD}=\frac{\mathrm{Registered}\,\mathrm{consumption}\,\mathrm{of}\,\mathrm{the}\,\mathrm{active}\,\mathrm{ingredient}\times 1,000\,\mathrm{inhabitants}}{\mathrm{Standard}\,\mathrm{DDD}\times \mathrm{no}.\mathrm{inhabitants}/\mathrm{period}\times 365\,\mathrm{days}}$$

To calculate this unit of measurement, firstly we considered as denominator the population undergoing active treatment for depression or anxiety. After that, we considered as denominator the total population of Aragon in the middle of each period.

Use of healthcare resources by these patients during the two study periods was assessed according to the use of PC services (number of nursing or general practitioner visits at the healthcare center or number of at-home healthcare visits and number of visits to other healthcare center professionals: physiotherapists, midwives, odontostomatologists, and social workers) and of hospital services [number of specialized care visits, number of diagnostic tests performed, number of visits to urgent care services, hospitalizations, and admission to intensive care units (ICU) and the duration of these stays].

### Statistical Analysis

The large sample size permits the use of parametric analysis methods (Lubin et al., 2005). Frequencies, means, and standard variations were used for the descriptive analysis of the study variables.

To determine variations in drug consumption, the DHD for each period and their annual equivalents were calculated. To compare the differences in the use of healthcare resources between the base measurement and the measurement taken 6 months following the lockdown, the paired Student’s *T*-test was used. For those variables with fewer number of observations than 100, Wilcoxon rank test was used. The level of significance was established at 5% (*p* < 0.05).

Statistical analysis was carried out using IBM SPSS Statistic 21 and R version 4.0.5.

### Ethical Considerations

The protocol followed for this study was approved by the Clinical Research Ethics Committee of Aragon (PI20–175). The procedures carried out for the creation of this work complied with the ethical standards of the previously mentioned committee and with the 1975 Declaration of Helsinki.

The processing, notification, and transfer of personal data were carried out in accordance with Regulation (EU) 2016/679 of the European Parliament and Organic Law 03/2018 on the Protection of Personal Data and the guarantee of digital rights.

## Results

Prior to the onset of the lockdown in Aragon, according to the ECR, 110,694 individuals over the age of 16 were undergoing active treatment for depression or anxiety. Of these, 5,104 (4.61%) were positive for COVID-19 infection during the study period. As for the number of deaths, 1,804 individuals passed away during this period: 4 during the 6 months prior to the onset of the lockdown, 242 during the at-home lockdown months, and 1,558 during the subsequent 6 months.

The sample, having a mean age of 61.7 years (16.9), consisted mainly of women (74.4%). Over 70% had an annual income of less than 18,000 euros (70.3%), and over 50% resided in urban areas (53.4%). Regarding to the presence of comorbidities, dyslipidemia (45.9%), hypertension (40.5%), back pain (35.9%), neoplasia (29.5%), and insomnia (20.0%) were the most frequent chronic conditions found in the study population (Table 1).

**TABLE 1:** Sociodemographic date and chronic comorbidities in patients suffering from anxiety or depression in Aragon.

	*N* (%)
**Sex**	
Men	28,294(25.6)
Women	82,400(74.4)
**Age**	
Mean (SD)	61.7 (16.9)
**Pharmaceutical service**	
<18,000	77,779(70.3)
Between 18,000–100,000	26,514(24.0)
>100,000	391 (0.4)
Free pharmacy	5,505(5.0)
Mutualist	474 (0.4)
Uninsured	31 (0.0)
**Basic health area**	
Urban	59,161(53.4)
Rural	51,533(46.6)
**Chronic comorbidities (yes %)**	
Arrhythmias	7,828(7.1)
Heart failure	3,343(3.0)
Ischemic heart disease	5,495(5.0)
Hypertension	44,884(40.5)
Dyslipidemia	50,792(45.9)
Obesity	15,621(14.1)
Overweight	2,432(2.2)
Disease in veins/arteries	3,091(2.8)
Cerebrovascular disease	6,613(6.0)
Diabetes	14,295(12.9)
Chronic bronchitis	1,592(1.4)
COPD	4,556(4.1)
Asthma	9,398(8.5)
Chronic kidney disease	7,403(6.7)
Hypothyroidism	16,011(14.5)
Hyperthyroidism	6,652(6.0)
Smoking	20,648(18.7)
Alcoholism	2,013(1.8)
Insomnia	22,105(20.0)
Autolytic attempt	1,075(1.0)
Anemia	20,372(18.4)
Neoplasia	32,657(29.5)
Dementia	4,034(3.6)
Hearing loss	9,911(9.0)
Cataracts	14,107(12.7)
Glaucoma	8,500(7.7)
Osteoarthritis	14,001(12.6)
Osteoporosis	16,882(15.3)
Back pain	39,788(35.9)

*SD, standard deviation; COPD, chronic obstructive pulmonary disease.*

Table 2 shows the variations in the consumption of drugs for the treatment of depression and anxiety among the population undergoing active treatment for these disorders and among general population. With respect to the pharmacological treatment of anxiety, in both groups there were a notable increase in the number of DHD of Alprazolam, Lormetazepam, and Lorazepam during the 6 months following the end of the lockdown. Other drugs, such as Triazolam or Brotizolam also experienced an increase in use among the population with depression and anxiety and among the general population, although to a lesser degree. With reference to anti-depressants, the number of DHD of both non-selective monoamine oxidase-inhibitors (MAO) and selective serotonin reuptake inhibitors (SSRIs), decreased as compared to the 6 months prior to the onset of the lockdown in both populations groups.

**TABLE 2:** Number of DHD 6 months before and 6 months after lockdown.

	DHD (patients suffering from anxiety or depression)		DHD (general population)	
	Six months before	Six months after	Six months before	Six months after
**Anxiolytics**				
Diazepam	14.22	14.19	1.18	1.18
Potassium clorazepate	3.00	2.98	0.25	0.25
Lorazepam	196.74	198.38	16.38	16.50
Bromazepam	3.30	3.26	0.27	0.27
Clobazam	0.16	0.16	0.01	0.01
Ketazolam	0.96	0.95	0.08	0.08
Alprazolam	305.61	316.80	25.44	26.35
Pinazepam	0.004	0.004	0.00	0.00
Bentazepam	0.005	0.000	0.00	0.00
**Hypnotics and sedatives**				
Flurazepam	0.20	0.20	0.02	0.02
Triazolam	2.35	2.48	0.19	0.21
Lormetazepam	452.00	459.21	37.63	38.19
Midazolam	0.26	0.28	0.02	0.02
Brotizolam	5.18	5.98	0.43	0.50
Quazepam	0.02	0.03	0.00	0.00
Loprazolam	4.15	3.90	0.34	0.32
**Antidepressants**				
Non-selective monoamine reuptake inhibitors				
Imipramine	0.01	0.01	0.00	0.00
Clomipramine	0.19	0.18	0.02	0.01
Trimipramine	0.00	0.00	0.00	0.00
Amitriptyline	0.50	0.49	0.04	0.04
Nortriptyline	0.04	0.03	0.00	0.00
Doxepin	0.00	0.00	0.00	0.00
Maprotiline	0.02	0.01	0.00	0.00
**Selective serotonin reuptake inhibitors**				
Fluoxetine	9.78	9.78	0.81	0.81
Citalopram	3.31	3.13	0.27	0.26
Paroxetine	12.98	12.49	1.08	1.04
Sertraline	5.40	5.39	0.45	0.45
Fluvoxamine	0.07	0.07	0.00	0.00
Escitalopram	60.82	58.45	5.06	4.86

As seen in Table 3, the number of ordinary and continuous PC nursing or general practitioner visits at the healthcare center or at-home visits were reduced during the 6 months after the end of the lockdown. Only the number of visits to the PC general practitioner at the healthcare center or by telephone increased during this same period [*p* < 0.001 (95% CI: −0.23 to −0.16)]. Similarly, the number of visits to other professionals at the healthcare center, such as midwives [*p* = 0.003 (95% CI: −0.72 to −0.14)], dentists [*p* = 0.033 (95% CI: −0.47 to −0.02)], and social workers [*p* = 0.001 (95% CI: −0.67 to −0.17)] increased; unlike the number of visits to physiotherapists, which experienced a notable decline [*p* < 0.001 (95% CI: 1.27 to 2.45)].

**TABLE 3:** Number of visits and diagnostic tests prescribed 6 months before and 6 months after lockdown.

		Mean (SD)		95% CI	*P*
	*N*	Six months before	Six months after		
Number of nursing visits (ordinary care) at health center or by telephone	45,428	3.91 (4.50)	3.56 (4.17)	0.31;0.39	<0.001
Number of nursing visits (ordinary care) at home	4,674	5.98 (8.47)	5.70 (8.48)	0.03;0.53	0.025
Number of nursing visits (continuous care) at health center	3,727	2.20 (3.21)	1.89 (2.77)	0.23;0.39	<0.001
Number of nursing visits (continuous care) at home	615	2.21 (2.90)	2.19 (4.63)	−0.37;0.42	0.903
Number of general practitioner visits (ordinary care) at health center or by telephone	92,640	5.98 (5.10)	6.18 (5.69)	−0.23;−0.16	<0.001
Number of general practitioner visit (ordinary care) at home	3,290	3.56 (3.82)	2.80 (3.19)	0.64;0.89	<0.001
Number of general practitioner visits (continuous care) al health center	9,358	2.06 (2.38)	2.07 (2.48)	−0.06;0.04	0.715
Number of general practitioner visits (continuous care) at home	743	1.80 (1.44)	1.66 (1.34)	0.02;0.26	0.022
Number of visits to other professionals					
Physiotherapist	678	6.30 (6.95)	4.44 (4.68)	1.27;2.45	<0.001
Midwife	584	2.35 (2.18)	2.79 (2.86)	−0.72;−0.14	0.003
Dentist	383	2.09 (1.67)	2.34 (1.77)	−0.47;−0.02	0.033
Social worker	653	2.63 (2.52)	3.06 (3.14)	−0.67;−0.17	0.001
Number of visits to specialized care (first consultation)	3,883	1.47 (0.85)	1.53 (0.92)	−0.10;−0.2	0.003
Number of visits to specialized care (successive consultations)	29,264	2.72 (2.32)	2.66 (2.41)	0.03;0.08	<0.001
Number of diagnostic tests performed					
X-rays	20,114	1.12 (1.23)	0.98 (1.19)	0.11;0.16	<0.001
Ultrasounds	20,114	0.30 (0.54)	0.28 (0.52)	0.01;0.03	<0.001
Resonances	20,114	0.14 (0.39)	0.15 (0.39)	−0.02;−0.01	0.010
CT scans	20,114	0.25 (0.57)	0.25 (0.57)	−0.01;0.00	0.178
Digestive tests	20,114	0.01 (0.11)	0.01 (0.10)	−0.00;0.00	0.921
Hemograms	22,451	0.30 (0.54)	0.26 (0.52)	0.03;0.05	<0.001
Biochemistry	22,451	1.01 (0.69)	0.90 (0.74)	0.10;0.12	<0.001
Microbiology	22,451	0.28 (0.67)	0.38 (0.79)	−0.11;−0.09	<0.001
Immunology tests	22,451	0.11 (0.35)	0.10 (0.34)	0.00;0.01	0.001
Coagulation	22,451	0.03 (0.19)	0.04 (0.20)	−0.01;−0.00	0.005
Urine tests	22,451	0.32 (0.59)	0.25 (0.56)	0.05;0.07	<0.001
Number of visits to A&E department	7,238	1.89 (1.67)	1.75 (1.49)	0.10;0.18	<0.001
Number of hospital admissions	6,413	1.27 (0.67)	0.28 (0.71)	0.96;1.00	<0.001
Number of days of hospital stays	1,230	16.66 (37.60)	17.11 (35.80)	−1.73;0.83	0.493
Number of ICU admissions	5	1.00 (0.00)	1.00 (0.00)		*
Number of days of ICU stays	5	108.80 (73.24)	100.80 (81.12)	−40.00;0.01	0.317^a^

*The correlation and t cannot be calculated because the standard error of the differences is 0. A&E, accident and emergency; ICU, intensive care unit; SD, standard deviation; 95% CI, confidence interval. ^a^Wilcoxon signed-rank test.

Regarding to the number of visits to specialists, the number of initial visits to these services increased as compared to the previous 6 months [*p* = 0.003 (95% CI: −0.10 to −0.2)]. On the other hand, the number of follow-up visits to these same services decreased [*p* < 0.001 (95% CI: 0.03 to 0.08)].

Diagnostic imaging (X-rays and ultrasounds) and laboratory testing (blood counts, bio-chemical analyses, and immunological tests) experienced a statistically significant decrease (*p* ≤ 0.001). There was also a statistically significant increase found in resonance and microbiological testing (*p* < 0.05).

Finally, visits to hospital services (number of visits to urgent care and number of hospitalizations), also decreased during the study period (*p* < 0.001).

## Discussion

The results of our study reveal variations in the total number of DHD dispensed in pharmacies for the pharmacological groups included in this study: N05B (anxiolytic drugs), N05C (hypnotics and sedatives), and N06A (antidepressants).

Regarding the increase observed in the total number of DHD of anxiolytic drugs (Alprazolam, Lormetazepam, and Lorazepam, mainly) during the 6 months following the onset of the lockdown; only one previous study, conducted in France on patients with depression, revealed similar results (Martinelli et al., 2021). Larger number of studies have analyzed the impact of the COVID-19 pandemic on the symptoms of patients having a past history of anxiety or depression. Individuals with prior mental illnesses appear to have had a higher risk or reporting increased levels of stress, anxiety, depression, impulsivity, anger, insomnias, or suicidal thoughts as compared to the general population (Hao et al., 2020; Liu et al., 2020). The increase in consumption of anxiolytic drugs shown in our study may be explained by the worsening of these symptoms (Martinelli et al., 2021). According to the Spanish Agency for Medicines and Medicinal Devices (AEMPS), at the end of 2019, the DHD of these drugs was 55.51. During the first quarter of 2020, it increased to 57.19, and by the fourth quarter of 2020, the consumption level peaked at 58.69 DHD (Agencia Española de Medicamentos y Productos Sanitarios (AEMPS), 2021). Spain, one of the hardest-hit countries during the first wave of the COVID-19 pandemic (Kontis et al., 2020), was already (before the healthcare crisis) ranked third amongst the European OECD (Organization for Economic Co-operation and Development) countries in terms of consumption of anxiolytic drugs, especially by women (Simó, 2018).

With respect to the minor decline in the number of DHD for anti-depressants, similar results were found in a study carried out in Portugal (Estrela et al., 2021). According to another study conducted in Aragon (Mestre, 2021), the follow-up period used for our study (6 months following the end of the lockdown) may be insufficient for the detection of changes in the consumption of these drugs. Immediately following the onset of the pandemic and throughout 2020, this study did not report any substantial changes in the prescribing of anti-depressants. As of 2021, however, an increased consumption became evident. This may be due to the continued pandemic situation.

The use of primary level care by these patients also declined during the months following the end of the lockdown. Previously published works (Firth et al., 2019) found interruptions in the care given to these patients as well as a redistribution of resources in response to the high number of positive COVID-19 cases. The increase observed in the number of PC visits to the general practitioner at the healthcare center or by telephone may be explained by the introduction of telemedicine, as a more effective alternative to in-person visits. Mental health is one of the specialties that best adapts to this tool, given that physical examination is not essential (Vieta et al., 2020).

The social and economic impact of the pandemic on the most vulnerable groups *a priori*, may be a cause of the increased social services demands (Grupo Estatal de Intervención en Emergencias Sociales, 2020). As for the increased number of visits to midwifes, although some studies (Ivanuš et al., 2021) suggest a decrease in the number of cervical cytology test during lockdown. The postponement of these visits to the midwife during the lockdown months might have led to an increased number of visits during the months following the end of this confinement period. In the same way, other studies (Davenport et al., 2020; Domínguez-Mejías et al., 2021) reveal an increase in symptoms of anxiety and depression amongst pregnant women during the pandemic months, which might have increased the number of these visits. Similarly might have occurred with the visits to the dentist. Postponing minor treatments may have led to larger problems (Elster and Parsi, 2021).

Finally, the increased number of initial visits to specialists may be a result of the declining physical health of these patients. Some studies (Van Rheenen et al., 2020) have revealed an increase in high-risk behaviors (alcohol consumption) during the pandemic months amongst individuals with previous mental illness; who usually have a higher number of comorbidities (obesity, diabetes, and cardiovascular illnesses) as compared to the general population (Firth et al., 2019), as well as higher prevalences of tobacco use (Prochaska et al., 2017).

The progressive increase in deaths is noteworthy. This increase, which may be the subject of future studies, may be related to the major impact of the first wave of the pandemic on Spain and its social and economic consequences. According to a recently published study (COVID-19 Mental Disorders Collaborators, 2021) in the most harshly hit countries (by the pandemic), an increased prevalence of depression and anxiety is anticipated. Both of these disorders and, even more so, the comorbidity between the two, are considered to be major risk factors in suicidal behavior (Moitra et al., 2021). The excess of mortality observed in our study during the 6 months following the end of the lockdown period may be explained in part by this fact. Statistics on suicide published in Spain in 2020 revealed an increase of 7.4% as compared to the prior year, a historical maximum (Fundación Española para la prevención del suicidio, 2021).

The results of our study reveal variations in the total number of DHD dispensed in pharmacies for the pharmacological groups included in this study: N05B (anxiolytic drugs), N05C (hypnotics and sedatives), and N06A (antidepressants).

Regarding the increase observed in the total number of DHD of anxiolytic drugs (Alprazolam, Lormetazepam, and Lorazepam, mainly) during the 6 months following the onset of the lockdown; only one previous study, conducted in France on patients with depression, revealed similar results (Martinelli et al., 2021). Larger number of studies have analyzed the impact of the COVID-19 pandemic on the symptoms of patients having a past history of anxiety or depression. Individuals with prior mental illnesses appear to have had a higher risk or reporting increased levels of stress, anxiety, depression, impulsivity, anger, insomnias, or suicidal thoughts as compared to the general population (Hao et al., 2020; Liu et al., 2020). The increase in consumption of anxiolytic drugs shown in our study may be explained by the worsening of these symptoms (Martinelli et al., 2021). According to the Spanish Agency for Medicines and Medicinal Devices (AEMPS), at the end of 2019, the DHD of these drugs was 55.51. During the first quarter of 2020, it increased to 57.19, and by the fourth quarter of 2020, the consumption level peaked at 58.69 DHD (Agencia Española de Medicamentos y Productos Sanitarios (AEMPS), 2021). Spain, one of the hardest-hit countries during the first wave of the COVID-19 pandemic (Kontis et al., 2020), was already (before the healthcare crisis) ranked third amongst the European OECD (Organization for Economic Co-operation and Development) countries in terms of consumption of anxiolytic drugs, especially by women (Simó, 2018).

With respect to the minor decline in the number of DHD for anti-depressants, similar results were found in a study carried out in Portugal (Estrela et al., 2021). According to another study conducted in Aragon (Mestre, 2021), the follow-up period used for our study (6 months following the end of the lockdown) may be insufficient for the detection of changes in the consumption of these drugs. Immediately following the onset of the pandemic and throughout 2020, this study did not report any substantial changes in the prescribing of anti-depressants. As of 2021, however, an increased consumption became evident. This may be due to the continued pandemic situation.

The use of primary level care by these patients also declined during the months following the end of the lockdown. Previously published works (Firth et al., 2019) found interruptions in the care given to these patients as well as a redistribution of resources in response to the high number of positive COVID-19 cases. The increase observed in the number of PC visits to the general practitioner at the healthcare center or by telephone may be explained by the introduction of telemedicine, as a more effective alternative to in-person visits. Mental health is one of the specialties that best adapts to this tool, given that physical examination is not essential (Vieta et al., 2020).

The social and economic impact of the pandemic on the most vulnerable groups *a priori*, may be a cause of the increased social services demands (Grupo Estatal de Intervención en Emergencias Sociales, 2020). As for the increased number of visits to midwifes, although some studies (Ivanuš et al., 2021) suggest a decrease in the number of cervical cytology test during lockdown. The postponement of these visits to the midwife during the lockdown months might have led to an increased number of visits during the months following the end of this confinement period. In the same way, other studies (Davenport et al., 2020; Domínguez-Mejías et al., 2021) reveal an increase in symptoms of anxiety and depression amongst pregnant women during the pandemic months, which might have increased the number of these visits. Similarly might have occurred with the visits to the dentist. Postponing minor treatments may have led to larger problems (Elster and Parsi, 2021).

Finally, the increased number of initial visits to specialists may be a result of the declining physical health of these patients. Some studies (Van Rheenen et al., 2020) have revealed an increase in high-risk behaviors (alcohol consumption) during the pandemic months amongst individuals with previous mental illness; who usually have a higher number of comorbidities (obesity, diabetes, and cardiovascular illnesses) as compared to the general population (Firth et al., 2019), as well as higher prevalences of tobacco use (Prochaska et al., 2017).

The progressive increase in deaths is noteworthy. This increase, which may be the subject of future studies, may be related to the major impact of the first wave of the pandemic on Spain and its social and economic consequences. According to a recently published study (COVID-19 Mental Disorders Collaborators, 2021) in the most harshly hit countries (by the pandemic), an increased prevalence of depression and anxiety is anticipated. Both of these disorders and, even more so, the comorbidity between the two, are considered to be major risk factors in suicidal behavior (Moitra et al., 2021). The excess of mortality observed in our study during the 6 months following the end of the lockdown period may be explained in part by this fact. Statistics on suicide published in Spain in 2020 revealed an increase of 7.4% as compared to the prior year, a historical maximum (Fundación Española para la prevención del suicidio, 2021).

Any study should be interpreted with caution. Our choice was to analyze the changes in behavior over a short period, between 6 months before and 6 months after lockdown, by collating the modification of the use of the care system and of anti-depressant and anxiety drugs. Starting from a postula: these increases reflect not only the increase in psychological suffering in these patients but also, they may be a proxy for the increase in prevalences of depression in general population. But it is relevant to highlight that this study uses an observational design, which does not allow for causal inferences. This is particularly important in the context of assessing COVID-19 lockdown effects, where multiple confounding factors may have influenced the outcomes.

Although the sample size was large, the mean age of the sample was high (61.7 years), and the age range of our sample was 87 years, with a minimum age of 16 years and a maximum age of 103 years. However, since real-world data (RWD) was used, all individuals who met the inclusion criteria were included. Therefore, this reflects the actual mean age of the population with depression in Aragon (Spain) before the start of the pandemic. The duration of the study may not be long enough to see variations in the severity of depression. The six-month comparison period may not be sufficient to capture long-term changes, especially in conditions such as depression that often require extended observation. This timeframe may have affected the detection of meaningful trends since depression is a disease that sets in gradually. While anxiety is subject to greater variability. But the significant increase in the use of anxiolytics is a warning sign. Studies should be continued to confirm or not the increase in the prevalence of depression, possibly expected thereafter.

Our source of information was a registry: the ECR. But this is not enough to provide objective information on the impact of the pandemic on the mental health of patients. To ensure the diagnosis of depression, the use of validated scales [Goldberg Anxiety and Depression Scale (GADS), Hamilton Anxiety Rating Scale, Beck Anxiety Inventory, etc.] will be necessary. This relates to the following limitation: the analysis did not adequately adjust for key confounders, such as differences in the severity of depression or anxiety, or changes in healthcare policies during the pandemic. Since there are no data on depression severity in the medical records, this factor could not be adjusted for. The absence of standardized assessments of symptom severity in electronic clinical records (ECRs) further limits the interpretation of outcomes. However, as there were no changes in the relevant medication guideline protocols issued by health authorities during the study period, it may be inferred that general practitioners tend to adjust medication upward in response to worsening symptoms of anxiety and depression, particularly following the initial titration period after a drug has been prescribed. Nevertheless, this cannot be definitively established based on the available data. Therefore, these findings should be interpreted with caution.

Finally, the number of statistical tests and calculated *p*-values in this article is large and therefore needs to be confirmed in further studies. A comparison at two time points of related samples (paired Student *T*-test) has been used, and for those variables with fewer number of observations than 100, Wilcoxon rank test was used. Some researcher consider that this may increases the risk of Type I errors.

## Conclusion

This study offers contributions, from a short-term perspective, with regard to the knowledge of the repercussions of lockdown measures on the use of drugs and health care resources, in a sample of patients undergoing active treatment for anxiety and/or depression, according to the ECR. However, the use of data from Electronic Clinical Records to investigate patients with anxiety and depression may have limitations.

The COVID-19 response should consider the mental health of the general population and should be especially cautious when considering more vulnerable groups, such as those with a past clinical history of depression or anxiety. Today, more than ever, it is necessary to invest in mental health services in an attempt to halt the anticipated growth of these disorders. Today, more than ever, it is necessary to invest in research and support measures for mental health services, to halt the anticipated growth of these disorders and the suffering in the population.

## Data Availability Statement

The data analyzed in this study is subject to the following licenses/restrictions: the data generated during the course of the project may be requested from the senior researcher of the project, in aggregate form. Requests to access these datasets should be directed to BO-B.

## Ethics Statement

The studies involving human participants were reviewed and approved by the Clinical Research Ethics Committee of Aragon (PI-20/175). Written informed consent from the participants’ legal guardian/next of kin was not required to participate in this study in accordance with the national legislation and the institutional requirements.

## Author Contributions

BO-B: conceptualization. SC-V, AC, BO-B, and AL-C: formal analysis. AL-C: writing—original draft preparation. AC, PN, BO-B, and SC-V: writing—review and editing. AC and BO-B: supervision. All authors have read and agreed to the published version of the manuscript.

## Conflict of Interest

The authors declare that the research was conducted in the absence of any commercial or financial relationships that could be construed as a potential conflict of interest.

## Publisher’s Note

All claims expressed in this article are solely those of the authors and do not necessarily represent those of their affiliated organizations, or those of the publisher, the editors and the reviewers. Any product that may be evaluated in this article, or claim that may be made by its manufacturer, is not guaranteed or endorsed by the publisher.
