# Correction: Analysis of drug and health resource use before and after COVID-19 lockdown in a population undergoing treatment for depression or anxiety

**DOI:** 10.3389/fpsyg.2025.1636749

**Published:** 2025-06-20

**Authors:** Ana Lear-Claveras, Ana Clavería, Sabela Couso-Viana, Patrice Nabbe, Bárbara Oliván-Blázquez

**Affiliations:** ^1^Institute for Health Research Aragón (IIS Aragón), Zaragoza, Spain; ^2^I-Saúde Group, South Galicia Health Research Institute, Vigo, Spain; ^3^Vigo Health Area, SERGAS, Vigo, Spain; ^4^Network for Research on Chronicity, Primary Care, and Health Promotion (RICAPPS), Barcelona, Spain; ^5^Département Universitaire de Médecine Générale, ER 7479 SPURBO (Soins Primaires, Santé Publique, Registre des Cancers de Bretagne Occidentale), Université de Bretagne Occidentale, Brest, France; ^6^Department of Psychology and Sociology, University of Zaragoza, Zaragoza, Spain

**Keywords:** COVID-19, depression, anxiety, quarantine, lifestyle, primary health care

In the published article, there were several instances where the text was incomplete. Additional wording has been included in certain parts of the text for added clarification.

Corrections have been made to **Discussion**, *Paragraphs 8, 9, 10 and 11*.

This text previously stated:

Any study should be interpreted with caution. Our choice was to analyze the changes in behavior over a short period, between 6 months before and 6 months after lockdown, by collating the modification of the use of the care system and of anti-depressant and anxiety drugs. Starting from a postula: these increases reflect not only the increase in psychological suffering in these patients but also, they may be a proxy for the increase in prevalences of depression in general population.

Although the sample size was large, the mean age of the sample was high (61.7 years). The duration of the study may not be long enough to see variations in the severity of depression. Depression is a disease that sets in gradually. While anxiety is subject to greater variability. But the significant increase in the use of anxiolytics is a warning sign. Studies should be continued to confirm or not the increase in the prevalence of depression, possibly expected thereafter.

Our source of information was a registry: the ECR. But this is not enough to provide objective information on the impact of the pandemic on the mental health of patients. To ensure the diagnosis of depression, the use of validated scales [Goldberg Anxiety and Depression Scale (GADS), Hamilton Anxiety Rating Scale, Beck Anxiety Inventory, etc.] will be necessary. In addition, it would be interesting to organize a qualitative research to complement this analysis. A cohort study using diagnostic tools to perform the sampling would be very useful in answering this question: are we on the cusp of an increase in depression in the general population due to the pandemic and which ones are the measures to deal with it?

Finally, the number of statistical tests and calculated *p*-values in this article is large and therefore needs to be confirmed in further studies.

The corrected paragraphs 8, 9, 10 and 11 are shown below.

Any study should be interpreted with caution. Our choice was to analyze the changes in behavior over a short period, between 6 months before and 6 months after lockdown, by collating the modification of the use of the care system and of anti-depressant and anxiety drugs. Starting from a postula: these increases reflect not only the increase in psychological suffering in these patients but also, they may be a proxy for the increase in prevalences of depression in general population. But it is relevant to highlight that this study uses an observational design, which does not allow for causal inferences. This is particularly important in the context of assessing COVID-19 lockdown effects, where multiple confounding factors may have influenced the outcomes.

Although the sample size was large, the mean age of the sample was high (61.7 years), and the age range of our sample was 87 years, with a minimum age of 16 years and a maximum age of 103 years. However, since real-world data (RWD) was used, all individuals who met the inclusion criteria were included. Therefore, this reflects the actual mean age of the population with depression in Aragon (Spain) before the start of the pandemic. The duration of the study may not be long enough to see variations in the severity of depression. The six-month comparison period may not be sufficient to capture long-term changes, especially in conditions such as depression that often require extended observation. This timeframe may have affected the detection of meaningful trends since *d*epression is a disease that sets in gradually. While anxiety is subject to greater variability. But the significant increase in the use of anxiolytics is a warning sign. Studies should be continued to confirm or not the increase in the prevalence of depression, possibly expected thereafter.

Our source of information was a registry: the ECR. But this is not enough to provide objective information on the impact of the pandemic on the mental health of patients. To ensure the diagnosis of depression, the use of validated scales [Goldberg Anxiety and Depression Scale (GADS), Hamilton Anxiety Rating Scale, Beck Anxiety Inventory, etc.] will be necessary. This relates to the following limitation: the analysis did not adequately adjust for key confounders, such as differences in the severity of depression or anxiety, or changes in healthcare policies during the pandemic. Since there are no data on depression severity in the medical records, this factor could not be adjusted for. The absence of standardized assessments of symptom severity in electronic clinical records (ECRs) further limits the interpretation of outcomes. However, as there were no changes in the relevant medication guideline protocols issued by health authorities during the study period, it may be inferred that general practitioners tend to adjust medication upward in response to worsening symptoms of anxiety and depression, particularly following the initial titration period after a drug has been prescribed. Nevertheless, this cannot be definitively established based on the available data. Therefore, these findings should be interpreted with caution.

Finally, the number of statistical tests and calculated *p*-values in this article is large and therefore needs to be confirmed in further studies. A comparison at two time points of related samples (paired Student *T*-test) has been used, and for those variables with fewer number of observations than 100, Wilcoxon rank test was used. Some researcher consider that this may increases the risk of Type I errors.

Additionally, a correction has also been made to **Conclusion**, *Paragraph 1*.

This paragraph previously stated:

This study offers contributions, from a long-term perspective, with regard to the knowledge of the repercussions of lockdown measures on the use of drugs and health care resources, in a sample of patients undergoing active treatment for anxiety and/or depression, according to the ECR.

The corrected paragraph is shown below.

This study offers contributions, from a short-term perspective, with regard to the knowledge of the repercussions of lockdown measures on the use of drugs and health care resources, in a sample of patients undergoing active treatment for anxiety and/or depression, according to the ECR. However, the use of data from Electronic Clinical Records to investigate patients with anxiety and depression may have limitations.

The authors apologize for these errors and state that these do not change the scientific conclusions of the article in any way. The original article has been updated.

